# Scalable Laser Processing Enables Transparent, Accretion Scale‐Independent, Ice‐Shedding Glass

**DOI:** 10.1002/advs.202524278

**Published:** 2026-03-12

**Authors:** Fan‐Wei Wang, Anish Pal, Arani Mukhopadhyay, Constantine M. Megaridis, Anish Tuteja

**Affiliations:** ^1^ Department of Chemical Engineering University of Michigan Ann Arbor Michigan USA; ^2^ Department of Mechanical and Industrial Engineering University of Illinois Chicago Chicago Illinois USA; ^3^ Macromolecular Science and Engineering Program University of Michigan Ann Arbor Michigan USA; ^4^ Department of Materials Science and Engineering University of Michigan Ann Arbor Michigan USA; ^5^ Biointerfaces Institute University of Michigan Ann Arbor Michigan USA

**Keywords:** fracture mechanics, ice shedding, ice/solid interfacial toughness, laser texturing

## Abstract

Ice accretion poses a formidable challenge for transparent surfaces in cold environments, such as windows, solar cells, and vehicle windshields. It is commonly understood that increasing surface roughness typically increases the forces required for the removal of ice from a substrate due to an increase in the ice‐solid interfacial area. We introduce a novel and scalable laser‐based technique to fabricate wave‐like micro‐patterns on glass surfaces, which defy this conventional understanding. Our results show that these patterns can strategically guide crack propagation at the ice‐glass interface, which significantly lowers the forces required for ice detachment, while preserving substrate transparency. Interestingly, once these micro‐patterns are present, variations in their amplitude and wavelength do not significantly impact the forces required for ice detachment. We present a comprehensive theoretical framework that explains these results, outlines the design principles for pattern fabrication to enable facile ice‐shedding in all directions, and support the model with experimental validation. Overall, this work offers a scalable strategy to create high‐performance, ice‐shedding glass and reassesses the role of surface roughness in facilitating passive ice detachment.

## Graphical abstract

We demonstrate a scalable laser‐based method to create wave‐like micro‐patterns on glass surfaces that counter the conventional view that roughness increases ice adhesion. These patterns direct crack propagation at the ice–glass interface, enabling low‐force, multi‐directional ice detachment while maintaining substrate transparency. A theoretical model and experiments confirm the underlying mechanisms and design principles.

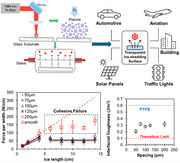

## 1 Introduction

Ice accretion presents a serious challenge across various industries, resulting in significant safety risks and economic losses [1]. In the aviation industry, icing can critically impair a plane's aerodynamics, contributing to 228 aircraft accidents between 2006 and 2010 [2]. Similarly, the buildup of ice on wind turbines in cold climates can cause annual power losses of approximately 20% [3]. Another common, yet persistent challenge is ice formation on transparent surfaces like windows, solar panels, and vehicle windshields [4, 5]. Traditional de‐icing methods for these surfaces often require active, energetically expensive solutions [6] or involve temporary coatings that need to be frequently reapplied [7]. Consequently, there is a pressing need for a passive, durable, and transparent solution that can effectively shed ice over both small and large accretion areas.

Passive deicing coatings [8], such as those that reduce the ice adhesion strength or delay ice formation, have recently gained significant attention. These coatings can reduce the energy demands of active systems considerably or, ideally, eliminate the need for energy input altogether. Prior work has even combined passive and active approaches by fabricating electrically conducting (and thus capable of Joule heating) superhydrophobic coatings [9]. A common metric used to evaluate a surface's ice‐shedding performance, or “icephobicity”, is the apparent ice adhesion strength (τ_*ice*_). τ_*ice*_ is defined as the force required to detach ice divided by the contact area between the ice and the substrate [10]. Surfaces with τ_*ice*_ <100 kPa are generally considered icephobic [11].

While τ_*ice*_ can be straightforward to measure, its interpretation requires caution due to the distinct mechanisms that may govern ice shedding. For example, a substrate may facilitate ice removal via interfacial slippage or interfacial fracture [12, 13]. In the case of interfacial slippage, ice slides on top of a slippery substrate (lubricated surfaces or mobile liquid‐like chains) and require continuous force to sustain the motion of ice on top of the substrate. In contrast, interfacial fracture involves detachment and permanent ice‐substrate separation once a critical force is reached, with no additional force required for ice motion.

Additionally, for interfacial fracture, the manner in which ice detaches from substrates can be scale‐dependent [14]. For shorter ice lengths (length is defined as the ice/substrate interface scale *along* the direction of applied force) or smaller interfacial areas, the entire ice block simultaneously separates from the substrate. The force per unit width required to cause this detachment $\tilde{F}$ linearly increases with the ice length *L*, following $\tilde{F}$ $=\hat{\tau }\;L$, where $\hat{\tau }$ is the ice adhesion strength. On the other hand, for longer ice lengths or larger interfacial areas, during ice detachment, a crack originates at one end of the block of ice and propagates to the other, leading to complete detachment. In this situation, the force per unit width (denoted as ${\tilde{F}}_{c}$) required for ice detachment becomes independent of the ice length, and its value is governed by the interfacial toughness Γ. These quantities can be related to one another via ${\tilde{F}}_{c}=\sqrt{2\Gamma E_{ice}h_{ice}}$. Here *E_ice_* =  8.5 *GPa* is the ice modulus and *h_ice_* is the ice thickness. Note that, for surfaces that utilize interfacial slippage, the force required to detach ice always increases linearly with ice length. Therefore, interfacial fracture might be more favorable for large‐scale de‐icing.

Numerous icephobic surfaces have been developed to mitigate ice accretion by reducing ice–substrate adhesion. Lowering surface energy reduces adhesion, but this approach has a lower limit of around 150 kPa [11]. Superhydrophobic surfaces can minimize interfacial contact by introducing air layers between the ice and the substrate, but frost formation can readily occur on such surfaces, leading to very high values of ice adhesion strength [15, 16]. Lubricated surfaces can yield low values for the effective ice adhesion strength but often suffer from poor durability due to progressive lubricant loss [17, 18]. Tuning the substrate mechanical properties offers an alternative that can be somewhat chemistry agnostic, though such surfaces also have some associated limitations. Soft, thick coatings promote interfacial cavitation, leading to low ice adhesion strength, but can lack mechanical durability [19, 20]. Composite coatings composed of hard matrices and soft fillers can balance low ice adhesion strength with improved mechanical durability [21, 22]. To further enhance deicing performance, an emerging approach incorporates the photothermal effect to supply additional heat for ice removal [23, 24, 25]. Overall, a variety of ice mitigation strategies have been developed thus far, but a single solution that works for all the different ice‐shedding applications does not yet exist. Therefore, identification of new ice‐shedding strategies and/or chemistries is crucial for developing effective solutions for specific ice‐affected applications.

Conventional understanding suggests that increasing surface roughness increases the forces required for ice detachment from a substrate due to an increase in the interfacial area. We challenge this notion here by utilizing a novel, scalable laser‐based technique that creates wave‐like micro‐patterns on glass. These patterns dramatically lower the forces needed for ice detachment by guiding crack propagation along the ice‐glass interface, all while maintaining the substrate's transparency. Our work also reveals a surprising finding: in many cases, once the texture patterns are present, variations in their amplitude and wavelength have no significant impact on the ice detachment forces. We discuss a comprehensive theoretical framework, validated by experimental measurements, to explain these unexpected results, and also provide design guidelines for pattern design to minimize the forces needed for ice detachment in all directions.

Our fabrication methodology employs a cost‐effective, readily available low‐power 1064 nm laser to texture the glass surfaces. Although previous studies have explored glass‐surface texturing, most of the methodologies have relied on lithography, stereolithography, masked etching, or costly ultra‐short pulsed lasers. All these approaches are either non‐scalable or lack precise control over surface microstructures [26, 27, 28, 29, 30, 31, 32, 33, 34]. In contrast, the laser ablation process introduced here is scalable, cost‐effective, and capable of achieving nanoscale control over surface morphology. The surfaces fabricated here remain highly transparent (transparency ∼92%) after texturing and once coated with a low‐surface‐energy silane layer, they demonstrate the unusual ice‐shedding properties described earlier. Note that although the low surface energy silane coating utilized here can bond with non‐textured glass and maintain its transparency [35], it alone is insufficient for facilitating the easy removal of ice from glass (details provided later).

## 2 Results and Discussion

### 2.1 Challenge for Ice Shedding on Glass: Design Principle of Periodic‐Structured Surfaces

Ice detachment is a thermodynamic process that occurs when the energy release rate at the ice‐substrate interface during fracture/delamination exceeds the interfacial toughness. This criterion can be expressed as $G\equiv -\frac{dU_{E}}{dA}\geq \Gamma$ [36, 37]. Here *G* is the energy release rate, which is quantified by the reduction in potential energy *U_E_* per crack opening area *A*. For ice detachment on rigid substrates like glass, the energy release rate can be approximated as $G=\frac{{\tilde{F}}^{2}}{2E_{ice}h_{ice}}$, where $\tilde{F}$ denotes the applied force per width. When $\tilde{F}$ reaches the critical value ${\tilde{F}}_{c}$, the energy release rate equals the interfacial toughness, giving $\frac{{{\tilde{F}}_{c}}^{2}}{2E_{ice}h_{ice}}=\Gamma$. Thus, lowering the interfacial toughness can be a feasible approach to reduce the forces required for ice detachment from glass.

The interfacial toughness represents the energy required to break the ice‐substrate interface, and includes the thermodynamic work of adhesion (*W_a_*), and the energy dissipated due to plastic flow (*E_p_*) [38, 39]. *W_a_* pertains to the energy difference between the ice‐substrate interface and the two new surfaces created after delamination and is influenced by both the substrate surface energy and roughness. Additionally, plastic deformation of the substrate can also absorb some energy during crack propagation. Overall, the interfacial toughness can be computed as

(1) $$\Gamma =r\left(\gamma_{ice}+\gamma_{sub}-\gamma_{ice-sub}\right)+E_{p}.$$

Here γ_*ice*_, γ_*sub*_, and γ_*ice* − *sub*_ are the surface energies for ice, the substrate, and the interfacial energy between ice and substrate, respectively, and *r* is the ratio between the actual area and the projected area (also known as the Wenzel roughness [40]).

**FIGURE 1:**
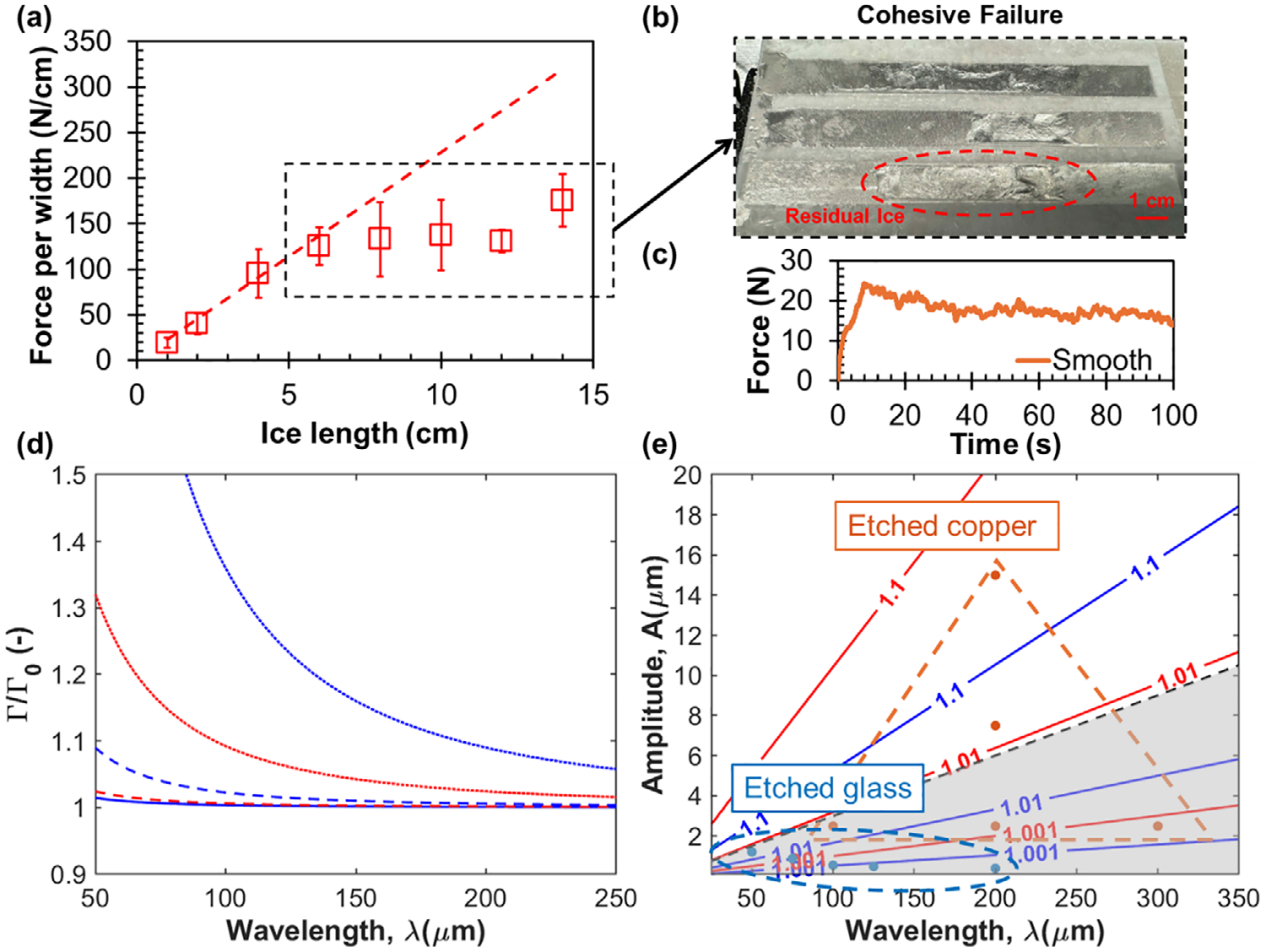
(a) Force per unit width vs. ice length curves for smooth, fluorinated glass. (b) An optical image highlighting the cohesive failure of ice on smooth glass at an ice length of 14 cm. Residual ice is clearly visible on the substrate after the push‐off test. (c) Force vs. time curves for smooth, fluorinated glass during an ice push‐off test at an ice length of 4 cm. (d) $\frac{\Gamma }{\Gamma_{0}}$ vs. wavelength for various texture amplitudes. The red color represents $\frac{\Gamma_{∥}}{\Gamma_{0}}$ and the blue color represents $\frac{\Gamma_{⊥}}{\Gamma_{0}}.$ The solid, dashed, and dotted lines respectively denote the amplitudes of 1, 2.5, and 10 µm. (e) Phase diagram for amplitude and wavelength as a function of the ratio between the actual interfacial toughness and the intrinsic interfacial toughness $\frac{\Gamma }{\Gamma_{0}}$. The red solid lines represent $\frac{\Gamma_{∥}}{\Gamma_{0}}$ for the case where the cracks propagate along the trenches. The blue, solid lines represent $\frac{\Gamma_{⊥}}{\Gamma_{0}}$ values for the case in which cracks propagate across the trenches. $\Gamma_{⊥}$ values in this figure are obtained from Equation 3. The blue dots denote the structural parameters for etched glass, while the orange dots denote the structural parameters for etched copper. The blue dashed ellipse and the orange dashed triangle, respectively, enclose the blue and orange dots to highlight their locations. The gray shadow represents the feasible range of amplitudes and wavelengths for low interfacial toughness.

Equation 1 suggests that lowering the substrate surface energy or roughness can reduce the interfacial toughness. Thus, intuitively, smooth (low roughness), fluorinated (low surface energy) glass should demonstrate good ice shedding performance. Here we grafted (heptadecafluoro‐1,1,2,2‐tetrahydrodecyl)‐dimethylchlorosilane on smooth glass (γ_*sub*_ ∼ 10 mN/m) to reduce its surface energy (see Supporting Information). Figure 1a shows the corresponding data for the force per unit width ($\tilde{F})$ as a function of ice length. The apparent ice adhesion strength for this modified glass is $\hat{\tau }$∼250 kPa, in line with previous measurements on similar hydrophobic surfaces [11, 14]. Perhaps more importantly, we observed cohesive failure for ice lengths longer than 4 cm (see Figure 1b). Cohesive failure occurs when strong ice‐substrate adhesion causes cracks to propagate through the ice, leaving residual ice on the surface, and this type of detachment is a clear indication of high adhesion between ice and the substrate.

The poor ice‐shedding performance of smooth, low surface energy glass likely arises from the high forces required for interfacial slippage on the surface. Figure 1c shows the force‐time response for a 4 cm‐long ice on the low‐surface‐energy silanized glass upon the application of a shear force (see Supporting Information). The finite forces required for the motion of ice at all times indicate that ice slips on the glass surfaces upon the application of a shear force, as opposed to fracturing cleanly. Since slippage lacks a toughness‐controlled regime, the required detachment force scales linearly with the contact length. At larger length scales (above ∼5 cm in this case), the required force to induce adhesive separation becomes larger than the force required for cohesive fracture, leading to poor ice removal from the substrate.

To develop glass with significantly lower ice adhesion, we fabricated parallel micro‐trenches (approximated as sinusoidal structures with amplitude A and wavelength λ) on the glass to guide crack propagation at the ice‐glass interface. We hypothesize that when *A* ≪ λ, the surface roughness will have a minimal impact on interfacial toughness. However, the periodic structure provided by the micro‐trenches may still facilitate crack propagation at the ice‐substrate interface.

To validate this hypothesis, we analyzed how surface amplitude *A* and wavelength *λ* impact the interfacial toughness Γ in parallel micro‐trenches. Crack orientation relative to the trenches is key to understanding this dependence. When cracks propagate across the micro‐trenches, their path is extended, increasing energy dissipation and setting an upper bound for Γ. Conversely, cracks propagating along the micro‐trenches avoid this path extension, defining the lower bound for Γ. The important design criterion to minimize Γ for the entire surface is to select values for *A* and *λ* that keep toughness near the inherent value of Γ for the surface, and consistent across different orientations. Note that the intrinsic value for Γ on a textured surface is the lowest interfacial toughness on the corresponding smooth surface with identical chemical composition, if ice‐shedding occurs in the interfacial fracture‐dominant regime.

For predicting the interfacial toughness for cracks propagating across the micro‐trenches, we can utilize the Cotterell–Rice approximation, which predicts the normalized interfacial toughness as [41]

(2) $$\frac{\Gamma_{⊥}}{\Gamma_{0}}={\left[\frac{2\sqrt{1+4\pi^{2}{\left(\frac{A}{\lambda }\right)}^{2}}}{1+\sqrt{1+4\pi^{2}{\left(\frac{A}{\lambda }\right)}^{2}}}\right]}^{2}\;,\;\mathrm{for}\;\frac{A}{\lambda }<0.25.$$

Here, $\frac{\Gamma_{⊥}}{\Gamma_{0}}$ is the ratio between the actual interfacial toughness $\Gamma_{⊥}$ when cracks propagate across the trenches to the intrinsic (no roughness) interfacial toughness $\Gamma_{0}$. Equation 2 suggests that interfacial toughness increases monotonically with A/λ, meaning that rougher surfaces yield higher toughness. However, this mode‐I fracture approximation for homogeneous materials may not fully capture the actual ice–glass interfacial behavior. To improve accuracy, finite element analysis (FEA) was used to model the relationship between $\frac{\Gamma_{⊥}}{\Gamma_{0}}$ and $\frac{A}{\lambda }$. The details of the simulation set‐up are provided in Supporting Information. By sampling different parameter sets and fitting the simulation results with a second‐order polynomial, we find that $\frac{\Gamma_{⊥}}{\Gamma_{0}}$ can be expressed as

(3) $$\frac{\Gamma_{⊥}}{\Gamma_{0}}=36{\left(\frac{A}{\lambda }\right)}^{2}+1.$$

On the other hand, when cracks propagate along the trenches, the increase in interfacial toughness mainly stems from the increase in the contact area. This increase in area can be quantified by the Wenzel roughness *r*. In this case [39, 42],

(4) $$\frac{\Gamma_{∥}}{\Gamma_{0}}=r=\frac{2}{\pi }\sqrt{1+4\pi^{2}{\left(\frac{A}{\lambda }\right)}^{2}}E\left(\frac{4\pi^{2}{\left(\frac{A}{\lambda }\right)}^{2}}{1+4\pi^{2}{\left(\frac{A}{\lambda }\right)}^{2}}\right)$$

Here Γ_∥_ is the actual interfacial toughness when cracks propagate along the trenches, and $E\;\left(m\right)=\int_{0}^{\frac{\pi }{2}}\sqrt{1-msin^{2}x}dx$ is the complete elliptic integral of the second kind. The detailed derivation for this relationship is provided in Supporting Information.

We plotted the normalized interfacial toughness values, $\frac{\Gamma_{∥}}{\Gamma_{0}}$ and $\frac{\Gamma_{⊥}}{\Gamma_{0}}$, as a function of the trench wavelength and amplitude in Figure 1d,e. These can help identify the desirable roughness values that can ensure low and orientation‐independent interfacial toughness values on the fabricated glass substrates.

Figure 1d shows that variations in both the amplitude and the wavelength of the trenches make a more significant impact on $\Gamma_{⊥}$ values as opposed to Γ_∥_. Additionally, $\Gamma_{⊥}$ and Γ_∥_ values decrease with either an increase in the trench wavelength or a decrease in the trench amplitude. When the trench amplitude is below ∼ 1 µm, and the wavelength is relatively large, the enhancements in $\Gamma_{⊥}$ and Γ_∥_ values when compared to $\Gamma_{o}$ are relatively small, and the $\Gamma_{⊥}$ and Γ_∥_ values are approximately equal. Therefore, trenches with large wavelengths and small amplitudes can be employed to achieve low, orientation‐independent interfacial toughness and thereby orientation‐independent forces for ice detachment.

To determine the feasible roughness regime that yields low interfacial toughness, we constructed a phase diagram mapping amplitude vs. wavelength, as shown in Figure 1e. Figure 1e shows that it is possible to design trenches that enable the facile shedding of ice in all directions. For instance, if the trench amplitude is A ∼ 2 µm, for all trench wavelengths λ > 100 µm, $\Gamma_{⊥}$ and Γ_∥_ values are likely to be within 1% of the intrinsic interfacial toughness. Similarly, for a trench amplitude of A ∼ 3 µm, all trench wavelengths λ > 150 µm are likely to yield similar values for $\Gamma_{⊥}$ and Γ_∥_ values. Combined with experimental data shown in the following sections, we suggest that low, orientation‐independent interfacial toughness can be achieved when $\frac{A}{\lambda }<0.03$ (the gray shadow region in Figure 1e).″

We validated these predictions by fabricating and testing the adhesion forces for ice detachment on low surface energy etched glass, as well as low surface energy roughened copper surfaces, as detailed below. The values of the amplitude and wavelength for the fabricated trenches on the different surfaces are also included in Figure 1d,e (blue points: etched glass; orange points: etched copper), and Table 1. As can be seen from Figure 1d,e, most values of A and λ were chosen in a manner to minimize differences in $\Gamma_{⊥}$ and Γ_∥_ values.

**TABLE 1:** Texture characteristics for the different glass and copper samples fabricated in this work, as measured by optical profilometry.

Glass			
Trench Spacing, λ (µm)	Wenzel Roughness, r (‐)	Amplitude, A (µm)	Amplitude to trench spacing ratio, A/λ (‐)
50	1.2	1.21	0.0242
75	1.14	0.88	0.0117
100	1.08	0.55	0.0055
125	1.04	0.49	0.0039
200	1.01	0.39	0.0020
Copper			
Trench Spacing, λ (µm)	Wenzel Roughness, r (‐)	Amplitude, A (µm)	Amplitude to trench spacing ratio, A/λ (‐)
100	1.09	2.5	0.025
200	1.04	2.5	0.013
300	1.04	2.5	0.008
200	1.12	7.5	0.038
200	1.40	15.0	0.075

### 2.2 Surface Structuring of Transparent Glass

**FIGURE 2:**
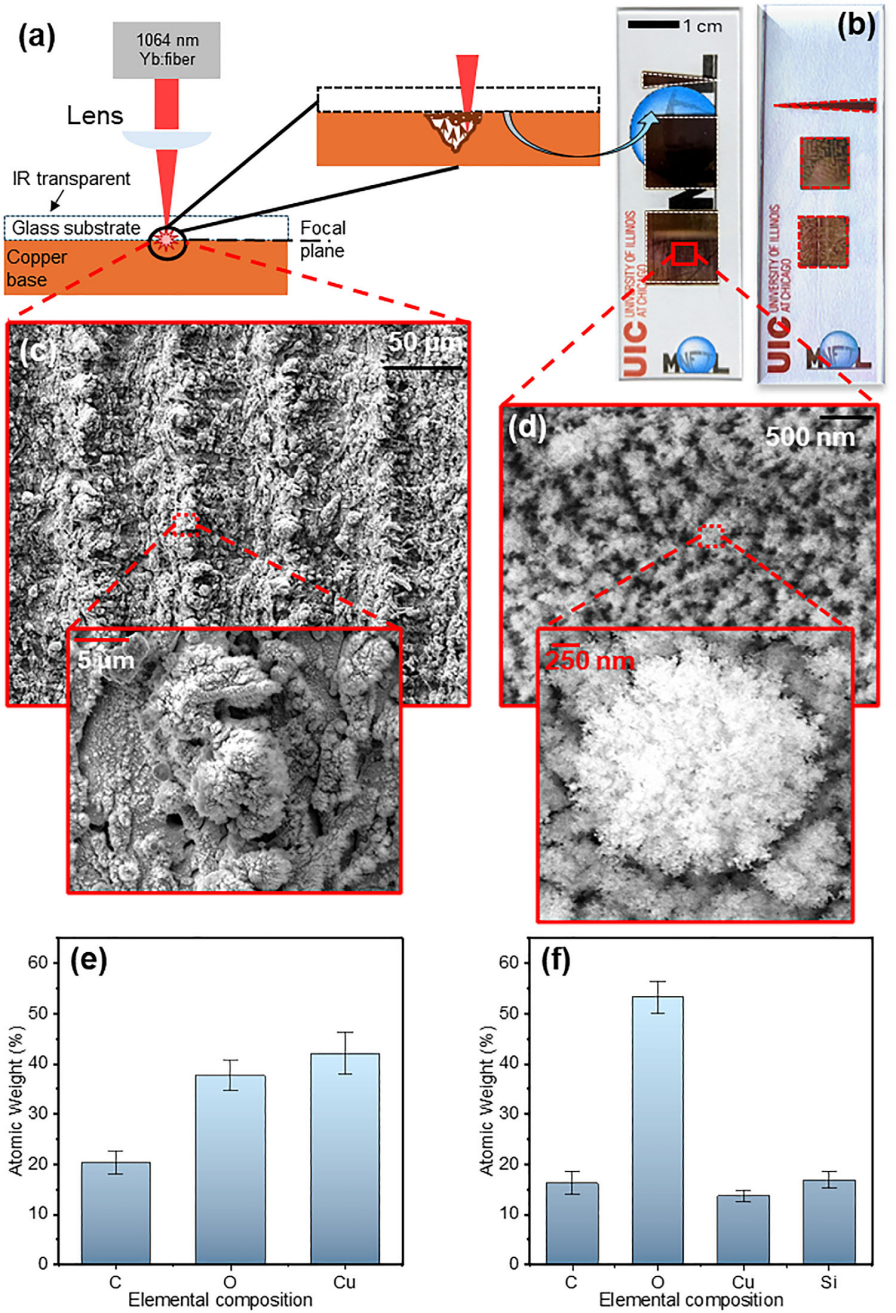
(a) Schematic depiction of the LIBT technique applied to a soda‐lime glass substrate with a 1064 nm Yb‐doped fiber laser source. (b) Copper deposition via LIBT on the underside of the glass substrate results in distinct opaque domains, while the surrounding glass remains transparent, as evidenced by the clearly visible text and symbols on the underlying paper. (c) SEM micrograph and magnified detail (inset) of the laser‐textured donor material (copper). (d) SEM micrograph along with a magnified inset showing the glass surface bearing light‐blocking copper deposits formed through the LIBT technique. (e), (f) Energy Dispersive Spectroscopy (EDS) respective analysis of the laser‐textured copper plate located beneath the glass surface and the glass surface bearing the LIBT‐deposited copper. The presence of copper and oxygen peaks validates the deposition of copper and its oxides on the underside of the glass slide.

To validate our ice‐shedding surface design, we developed a novel, scalable, laser‐etching technique to create micro‐trenches on glass. Soda‐lime glass surfaces were initially sonicated in acetone for 30 min to eliminate any surface contaminants potentially introduced during handling or sample transport, followed by drying with ultra‐high purity nitrogen gas (bottled 99.999%). Glass texturing was performed using a near‐infrared fiber laser operating at 1064 nm (Tykma Electrox EMS 300). Since glass is transparent to infrared (IR) radiation, it cannot be directly textured using infrared lasers, as is possible with infrared‐absorbing materials [43]. To overcome this, a two‐step processing approach was implemented. Initially, laser‐induced backward transfer (LIBT) was employed, as illustrated in Figure 2a, using copper as the donor material. During the LIBT process, the IR laser beam passes through the transparent glass substrate and ablates the underlying infrared‐absorbing donor layer (copper, in this case), resulting in the metal being deposited onto the adjacent bottom surface of the glass. The deposition process simultaneously induces surface roughness on the underside of the glass in contact with the copper layer. As shown in Figure 2b, copper domains of varying shapes can be transferred onto the overlaying surface of the glass. Figure 2c displays a scanning electron microscope (SEM) micrograph of the copper plate that was textured by the laser beam transmitted through the infrared‐transparent glass substrate. Figure 2d shows an SEM image of the copper deposited on the underside of the glass. Figure 2e,f present elemental compositions obtained from energy‐dispersive spectroscopy (EDS) spectra corresponding to the laser‐textured copper surface and the glass surface with deposited copper, respectively.

The detection of copper and oxygen in Figure 2f confirms the successful deposition of copper on the underside of the glass slide. Additionally, as laser processing was conducted in ambient atmospheric conditions, the surface structuring not only resulted in copper texturing but also induced metal oxidation due to the thermal energy involved. This is evidenced by the presence of oxygen peaks in both graphs presented in Figure 2e,f.

**FIGURE 3:**
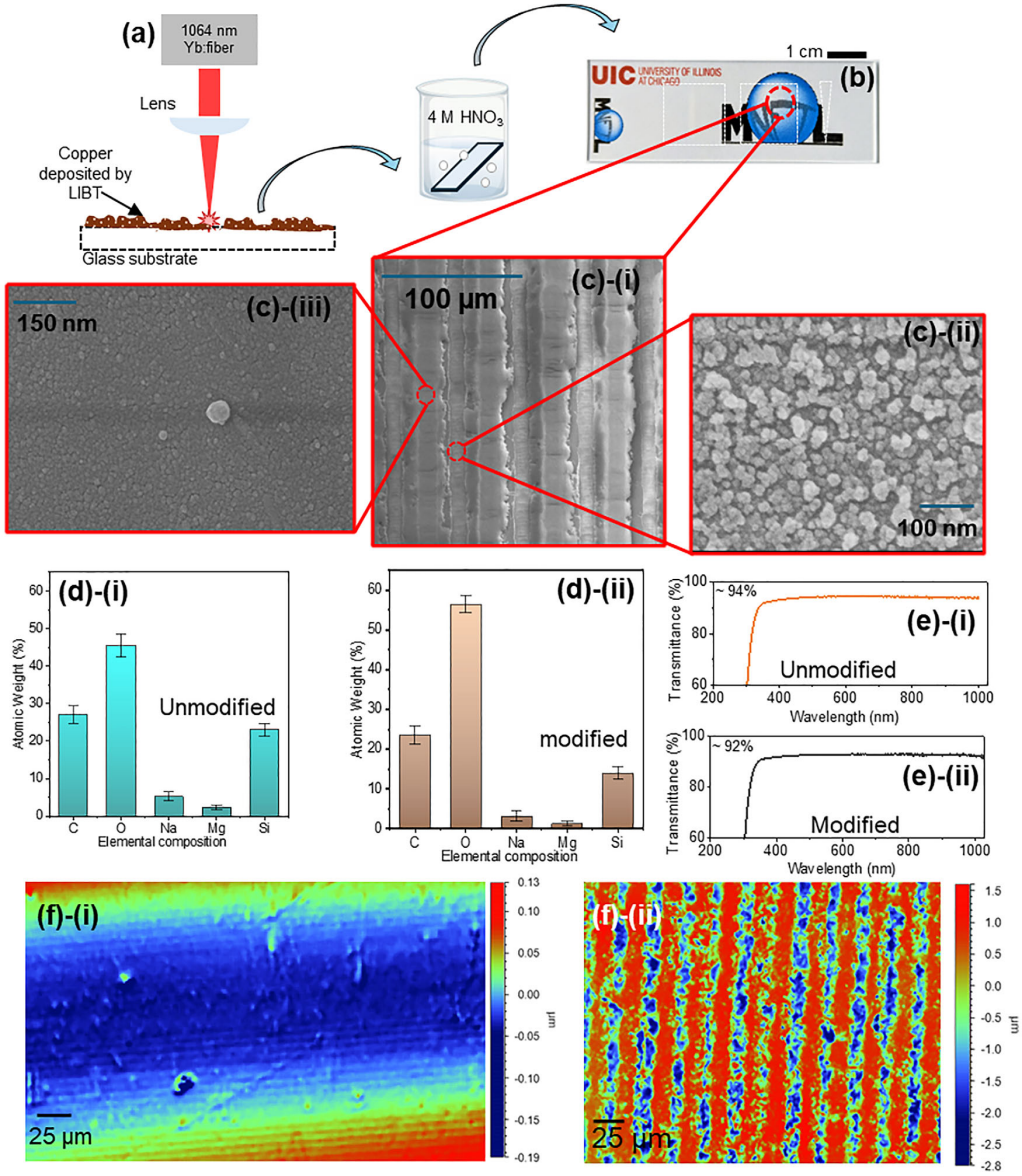
(a) Schematic depiction of laser ablation of copper‐coated glass surface to render the surface transparent. (b) Acid‐cleaned transparent glass with localized texturing, revealing underlying logos on the paper placed beneath the glass slide. (c) (i): SEM image of a laser‐textured (trench spacing 50 µm) transparent glass surface after copper removal and cleaning, (ii): SEM image (magnified) of the laser‐ablated region on the glass surface, (iii): SEM image (magnified) of the unablated region on the glass surface. (d) (i),(ii) Respective EDS elemental plots of unprocessed and laser‐textured glass surface showing no chemical decomposition after laser texturing. (e) (i), (ii) Respective transparency plots of unprocessed and laser‐textured glass surface showing a negligible dip (2%) in transparency. (f) (i),(ii) Respective optical profilometry maps of unprocessed and processed glass surfaces showing the texture imparted through laser processing.

Following the LIBT process, the treated surface becomes opaque to infrared (and visible) light due to copper deposition and its swift oxidation during laser ablation (See Figure 2b). In the second processing step, the glass plate is flipped to expose the deposited copper layer, which is then removed through laser ablation, as illustrated in Figure 3a. In this step, the laser beam (50 µm dia.) ablates the surface along raster lines spaced 50–200 µm apart and parallel to the length of the slide. Relevant laser parameters employed for the LIBT process and the subsequent copper removal process can be found in Table S1. Following the ablation step, the glass is sonicated first in a 4 m nitric acid (HNO_3_) aqueous solution (15 mins) and then in ethanol (15 mins) to eliminate any residual debris. The thermal energy transferred from the thin copper film to the underlying glass during copper deposition and removal—when performed under appropriate conditions—causes localized heating and melting of the glass (detailed laser parameters are provided in Supporting Information). The subsequent recrystallization results in a roughened, yet‐transparent glass surface. Figure 3b shows the transparent, textured glass surface after copper removal. SEM micrographs of the roughened glass surface are presented in Figure 3c(i–iii), with Figure 3c(ii,iii) highlighting the ablated and unablated regions, respectively. The corresponding EDS bar graphs in Figure 3d(i,ii) confirm that the laser texturing process did not lead to any elemental alteration or degradation of the glass composition. Despite the surface roughening achieved through this engineering approach, the glass retains a high level of transparency (∼92%), as shown by the transmittance curves in Figure 3e, which indicate that the laser texturing causes only a minimal reduction in optical clarity.

The optical profilometry maps in Figure 3f(i,ii) compare the surface profiles of untextured and laser‐textured glass, respectively. The profile of the textured surface (see Figure 3f(ii)) reveals the micro‐ and nanoscale roughness imparted from the laser ablation process. Also see Table 1 for additional profilometry data.

### 2.3 Ice Shedding Performance

The design principles outlined in Section 2.1 suggest that low texture amplitudes combined with large trench spacings are expected to yield low and orientation‐independent interfacial toughness for ice. To validate this prediction, we employed the laser‐etching technique described in Section 2.2 to systematically vary the trench spacing, while maintaining minimal surface roughness (*r* < 1.2). Next, the surfaces were silanized, following which we measured the adhesion strength of ice on the fabricated surfaces at different ice accretion lengths (see Figure S5 for silanization details and ice adhesion testing set‐up). Silanization lowers the surface energy, thereby reducing ice adhesion in accordance with Equation 1. Similar advancing (∼120°) and receding (∼90°) contact angles across all spacings (see Figure S6) indicate that water droplets (and likely ice) remain in the Wenzel state, with no air gap at the ice–substrate interface [15, 44]. Consequently, variations in ice adhesion primarily arise from differences in surface roughness or spacing, rather than changes in the ice‐substrate contact area.

**FIGURE 4:**
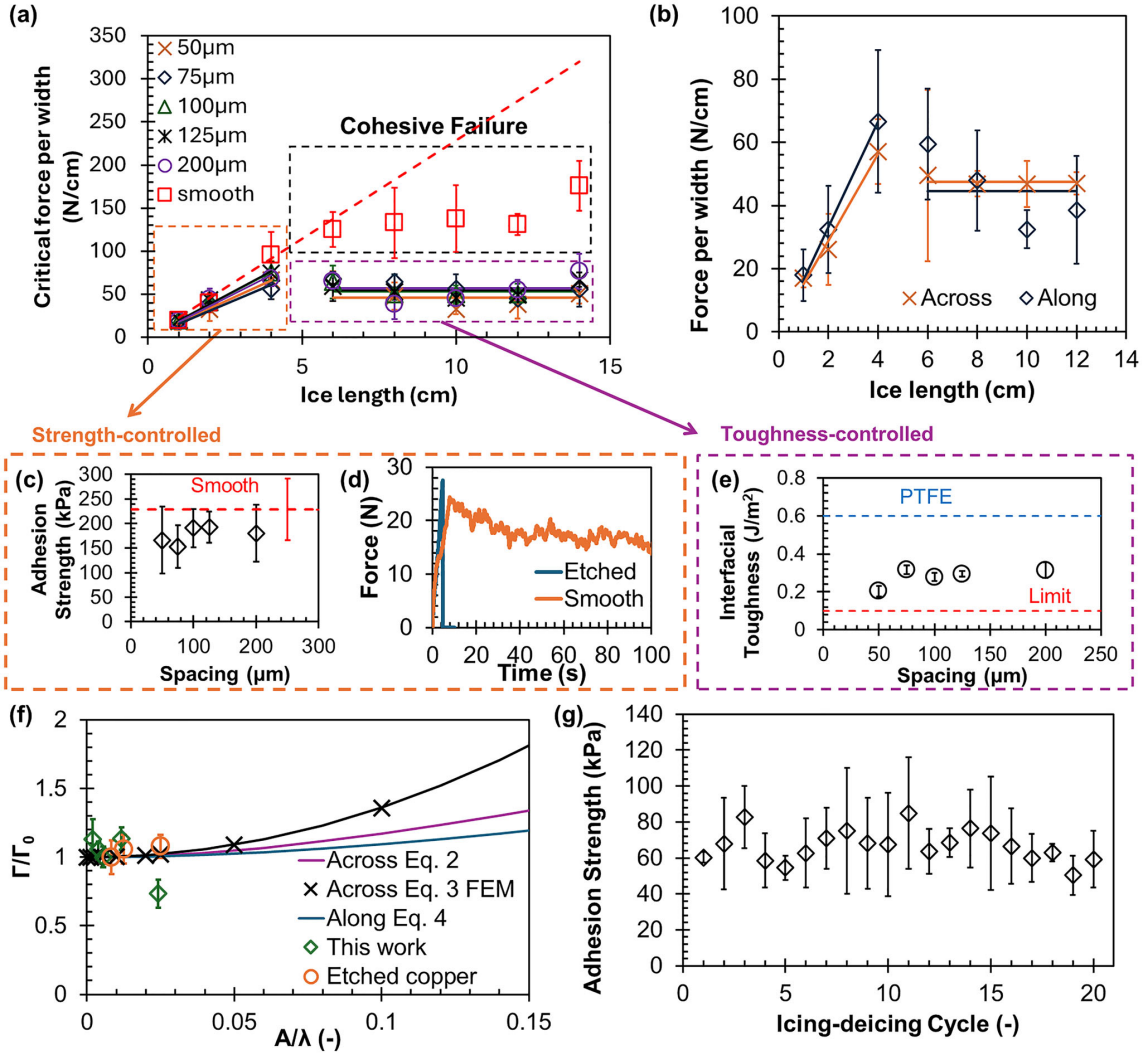
Ice shedding performance of hydrophobized, laser‐etched glass. (a) Measured force per unit width vs. ice length values for smooth glass and etched glass with different roughness trench spacings. (b) Measured force per unit width vs. ice length values for etched glass with trench spacing of 50 µm with different ice/roughness relative orientations. “Across” denotes that cracks propagate across multiple trenches. “Along” denotes that cracks propagate along the trenches. (c) The relationship between ice adhesion strength and trench spacing for etched glass. The red dashed line represents the ice adhesion strength for smooth glass (no trenches). (d) Typical force vs. time curves for smooth and etched glass, measured during ice push‐off experiments. (e) The relationship between ice/glass interfacial toughness and trench spacing. The red dashed line is the theoretical limit, and the blue dashed line is the interfacial toughness of PTFE. (f) Relationship between normalized interfacial toughness and $\frac{A}{\lambda }$. The green diamonds, orange circles, and black crosses represent etched glass (this work), etched copper, and FEA results, respectively. (g) Durability performance of etched glass. Here, we used etched glass with a trench spacing of 100 µm as an illustrative example. The error bars denote standard deviations obtained from at least four different measurements under identical conditions.

Figure 4a–e compares the ice shedding performance of laser‐etched and smooth glass at various ice lengths and a fixed width of 1 cm. At shorter ice lengths (L ≤ 4 cm), smooth glass exhibits slightly higher adhesion strength than all the fabricated etched glass surfaces (A = 0.39–1.21 µm; λ = 50–200 µm) (Figure 4a). Despite somewhat similar adhesion values, the detachment behavior between the smooth and textured glass surfaces differs significantly. On the textured glass, ice undergoes distinct fracture and clean separation, as indicated by a sharp rise and sudden drop in the force‐time curve (Figure 4d). In contrast, ice on the smooth glass exhibits slippage under applied force. As such, the ice moves on top of the smooth glass surface upon the application of the shear force, but does not detach. Consequently, ice requires continuous force to maintain motion. This suggests that the fabricated micro‐trenches can successfully induce interfacial fracture.

For longer ice lengths, the textured glass surfaces require significantly lower forces for ice detachment when compared to smooth glass, an outcome that appears counterintuitive and thus deserves special attention. On smooth glass, ice fails cohesively, leaving residual ice behind. In contrast, ice removal from etched glass is via interfacial fracture, and the forces for detachment are governed by interfacial toughness. As such, above a critical length of ∼5 cm, the forces for ice detachment are low and independent of ice length (Figure 4a). For all the etched glass surfaces, we measured a low interfacial toughness value of a Γ ≈ 0.3 J/m^2^. This interfacial toughness value is approximately half the value for PTFE (Γ ≈ 0.6 J/m^2^, a commercial low surface energy polymer which has previously been shown to have a very low interfacial toughness with ice) and approaches the theoretical minimum value of interfacial toughness for ice of Γ ≈ 0.1 J/m^2^ [14].

Notably, trench amplitude and wavelength (see Figure 4e), as well as the relative orientation between ice and the trenches (see Figure 4b) had little effect on measured forces for ice detachment. These measurements show that: (1) our model can be utilized effectively to guide the design of materials with low, orientation‐independent interfacial toughness; and (2) introducing trenches with an amplitude as low as 0.39 µm is sufficient to significantly reduce the forces required for ice detachment, and shift the mode for ice shedding on glass from cohesive failure to toughness‐controlled interfacial fracture. Notably, we also applied a different silane (1,3‐dichlorotetramethyldisiloxane) to the glass substrates and observed similarly low, orientation‐independent interfacial toughness (Γ ≈ 0.3 J/m^2^, see Figure S7). This result further supports the broad applicability of our proposed design framework and the flexibility in material selection accorded by this approach for different applications.

To evaluate if the developed approach can aid the shedding of ice from other surfaces besides glass, we also fabricated low‐surface‐energy, etched copper substrates (see Supporting Information for fabrication details and Figure S8 for substrate morphology and ice shedding performance). The trench amplitudes and wavelengths for etched copper were varied over a wider range than for glass (A = 2.5–15.0 µm; λ = 100–300 µm).

Figure 4f plots the interfacial toughness values as a function of the trench amplitude to wavelength ratio A/λ for both the textured glass and copper surfaces. The experimental data show good agreement with both the analytical model (Equations 2 and 4), as well as FEA simulations (Equation 3) for both glass and copper. The FEA results predict higher values of interfacial toughness than the Cotterell‐Rice approximation, though the deviations between the model and simulations only become significant at larger $\frac{A}{\lambda }$ values ($\frac{A}{\lambda }>0.03)$. Most of the surfaces fabricated in our work had $\frac{A}{\lambda }$ values < 0.03.

Apart from large $\frac{A}{\lambda }$ values, plastic deformation during crack propagation can also increase the forces required for ice detachment beyond what is predicted from our model. For example, the experimental results for etched copper show that cohesive fracture happens for $\frac{A}{\lambda }>0.03$ (Figure S8b). Regardless, trenches with low amplitude and high wavelength ($\frac{A}{\lambda }<0.03$) can be utilized to readily fabricate surfaces that enable the facile shedding of ice across all orientations. On the other hand, for larger trench amplitudes and/or smaller trench wavelengths, the forces for ice detachment can increase significantly beyond the values for a smooth surface, even leading to the cohesive failure of ice. Finally, to confirm the long‐term usability and reproducibility of the developed samples, we measured ice‐adhesion strength across multiple samples (of the same type) and multiple spots on the same sample. We also tested the ice‐adhesion strength on the same spot over multiple icing‐deicing cycles. This data is shown in Figure 4g. From the data, it is clear that the measured ice adhesion strength on our samples remains nearly unchanged after 20 icing‐deicing cycles, demonstrating the promise of the developed samples for long‐term real‐world applications.

## 3 Conclusion

We have introduced a new theoretical framework that demonstrates how carefully controlled, wave‐like micro‐patterns can guide crack propagation at the ice‐substrate interface, leading to a significant and orientation‐independent reduction in ice adhesion when compared to a smooth surface with the same chemical composition. Additionally, the model predicts that while the presence of these patterns is essential for lowering ice adhesion, variations in their specific amplitude and wavelength may have a negligible impact on the required ice detachment forces. To experimentally validate these counterintuitive predictions, we developed a scalable laser‐etching technique to create wave‐like micro‐trenches on transparent glass. Once silanized to lower surface energy, the resulting transparent material matched the model predictions and demonstrated excellent ice‐shedding properties with an extremely low ice‐substrate interfacial toughness of ∼0.3 J/m^2^.

## Conflicts of Interest

The authors declare no conflicts of interest.

## Supporting information




**Supporting File**: advs74744‐sup‐0001‐SuppMat.docx.

## Data Availability

The data that support the findings of this study are available in the supplementary material of this article.
