# Supplementary material for: Scalable Laser Processing Enables Transparent, Accretion Scale‐Independent, Ice‐Shedding Glass
Source: Adv Sci (Weinh). 2026 Mar 12;13(30):e24278. doi: 10.1002/advs.202524278 (PMC13248785; doi:10.1002/advs.202524278)
Supplement: Supplementary file 1 — Supporting File: advs74744‐sup‐0001‐SuppMat.docx. [file ADVS-13-e24278-s001.docx]

**Supporting Information**

Scalable Laser Processing Enables Transparent,
Accretion Scale -Independent Ice-Shedding Glass

*Fan-Wei Wang^1^, Anish Pal^2^,* *Arani Mukhopadhyay^2^,* *Constantine Megaridis^2,*^, Anish Tuteja^1,3,4,5,*^*

^1^ Department of Chemical Engineering, University of Michigan, Ann Arbor, MI, USA

^2^ Department of Mechanical and Industrial Engineering, University of Illinois Chicago, Chicago, IL, USA

^3^ Macromolecular Science and Engineering Program, University of Michigan, Ann Arbor, MI, USA

^4^ Department of Materials Science and Engineering, University of Michigan, Ann Arbor, MI, USA

^5^ Biointerfaces Institute, University of Michigan, Ann Arbor, MI, USA

*Corresponding authors. Email: Anish Tuteja (atuteja@umich.edu) or
 Constantine M. Megaridis (cmm@uic.edu)

*Fabrication of transparent rough glass*

Laser texturing of glass

**Table S1** presents the laser parameters employed for both the laser-induced backward transfer (LIBT) process and the subsequent removal of copper from the glass surfaces. As seen in this table, the laser beam rastering was done so that the trenches along the length of the glass slide matched for both the LIBT and the copper-removal scans. However, the beam power was three times as strong in the copper-removal scans.

| **Table S1: Laser parameters (Raster velocity 200 mm/s; Pulsing frequency 20 kHz)** | | | | |
| --- | --- | --- | --- | --- |
| **LIBT** | | | **Cu Removal** | |
| **Sample** | **Line Spacing (µm)** | **Power (W)** | **Line Spacing (µm)** | **Power (W)** |
| 1 | 50 | 6 | 50 | 18 |
| 2 | 75 | 6 | 75 | 18 |
| 3 | 100 | 6 | 100 | 18 |
| 4 | 125 | 6 | 125 | 18 |
| 5 | 200 | 6 | 200 | 18 |

Textured surface microstructure

**Figure S1** shows the microstructure of the laser-textured glass surface *before* nitric acid cleaning. At smaller line spacings (**Fig. S1(a–c)**), both the hills and trenches exhibit dense residual metal deposits, which originate from redeposition of ablated material from neighboring trenches due to strong scan overlap and localized thermal accumulation. As the line spacing increases (**Fig. S1(d,e)**), the redeposition effect diminishes, resulting in progressively sparser and eventually negligible residual metal on the surface. These observations highlight the strong dependence of metal redeposition on raster-line spacing during the LIBT and subsequent laser-ablation processes.


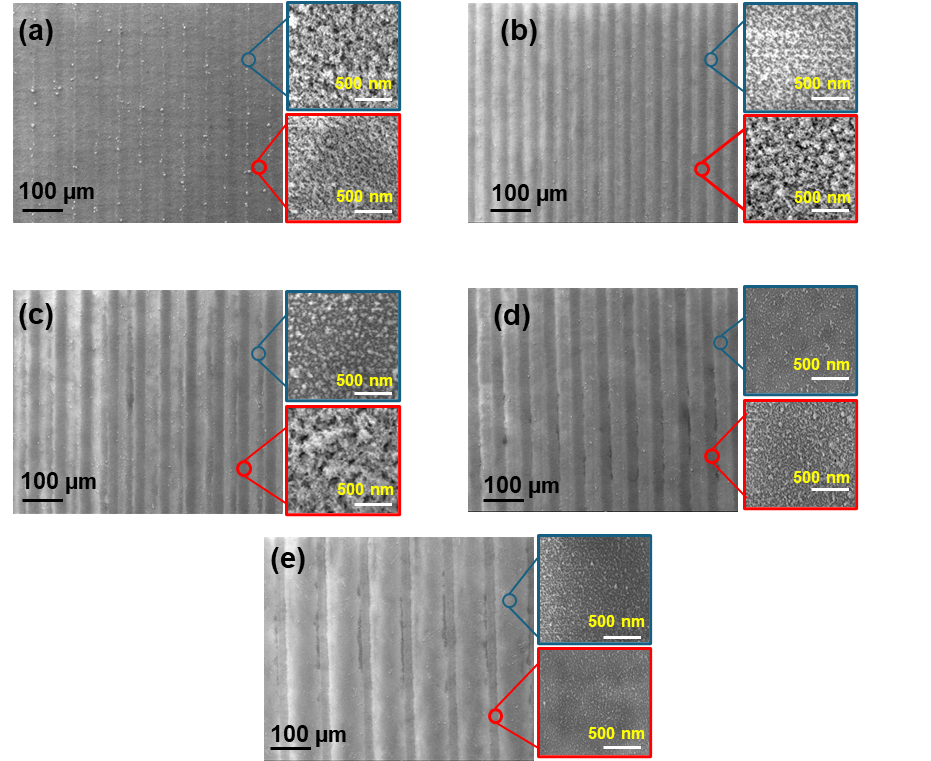


**Figure S1:** (a)-(e): Scanning electron micrographs of surfaces 1-5 (Table S1), respectively, *before* nitric acid cleaning. The red-bordered insets are magnified details at locations exposed to the metal-removing beam (second ablation step). The blue-bordered insets show surface details at locations not exposed to the metal-ablating beam.

**Figure S2** depicts the scanning electron micrographs (SEMs) of four different glass sample surfaces after copper removal and cleaning; Samples 2-5 in **Table S1** *after* nitric acid cleaning. All residual metal has been removed, and only pure glass microstructure is visible in these images.


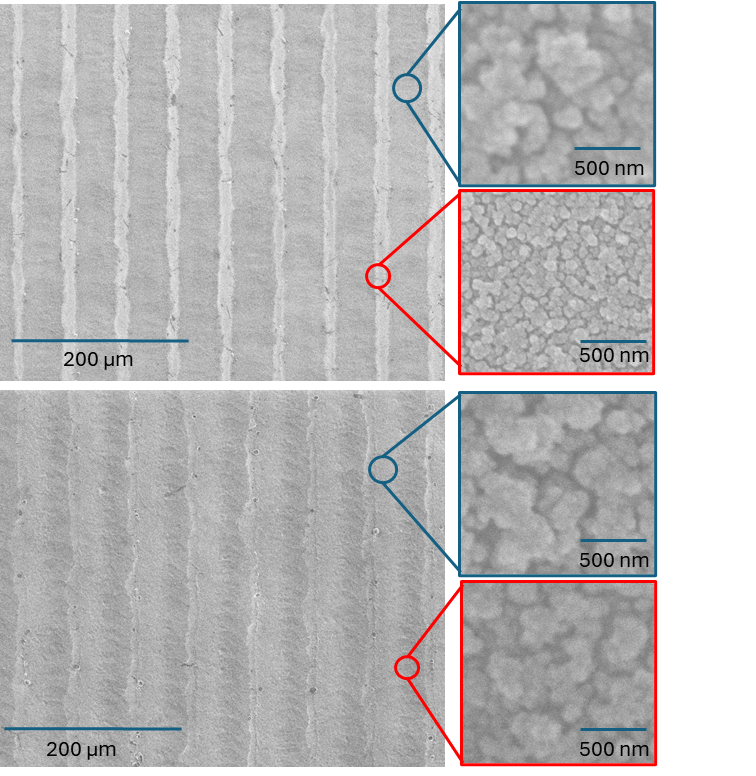

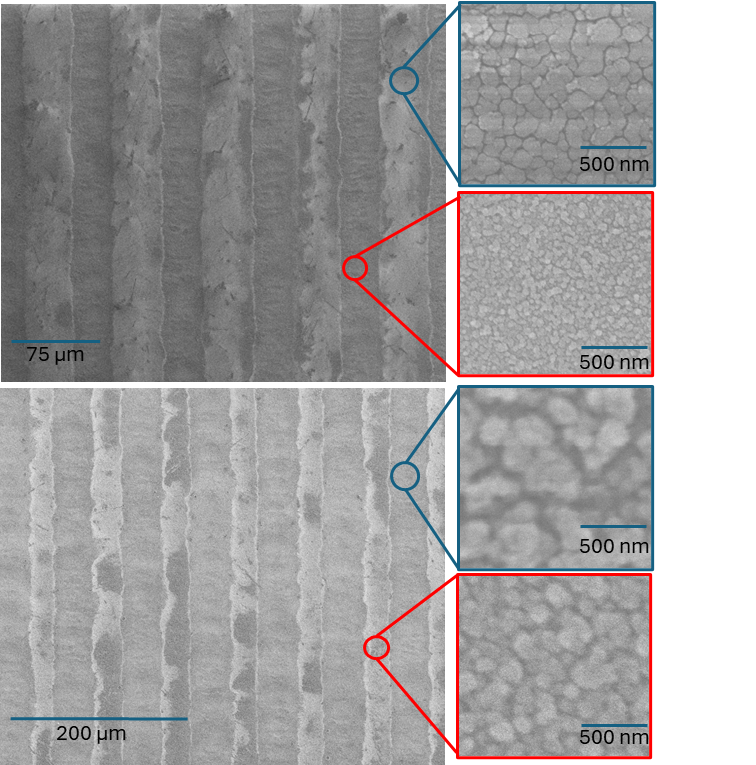


**(a)**

**(b)**

**(c)**

**(d)**

**Figure S2:** (a)-(d): Scanning electron micrographs and magnified inset details of surfaces 2-5 (Table S1), respectively, *after* nitric acid cleaning. An SEM image of sample surface 1 is in **Fig. 3c** of the main manuscript.

Transparency before Acid Cleaning

**Figure S3** shows the optical transmittance spectra of surfaces S1 and S5 in the visible wavelength range prior to nitric-acid cleaning. The higher-textured surface (S1) exhibits lower transparency than S5, attributable to residual metal present in both the hills and trenches of the laser-processed glass microstructure, which enhances light scattering and absorption. With the nitric-acid cleaning step, all residual metal is removed, resulting in substantial recovery of optical transparency, with transmittance ~ 92%.

**Figure S3**: Transparency plots of surfaces S1 and S5 *before* nitric-acid cleaning.

Surface Texture Profiles


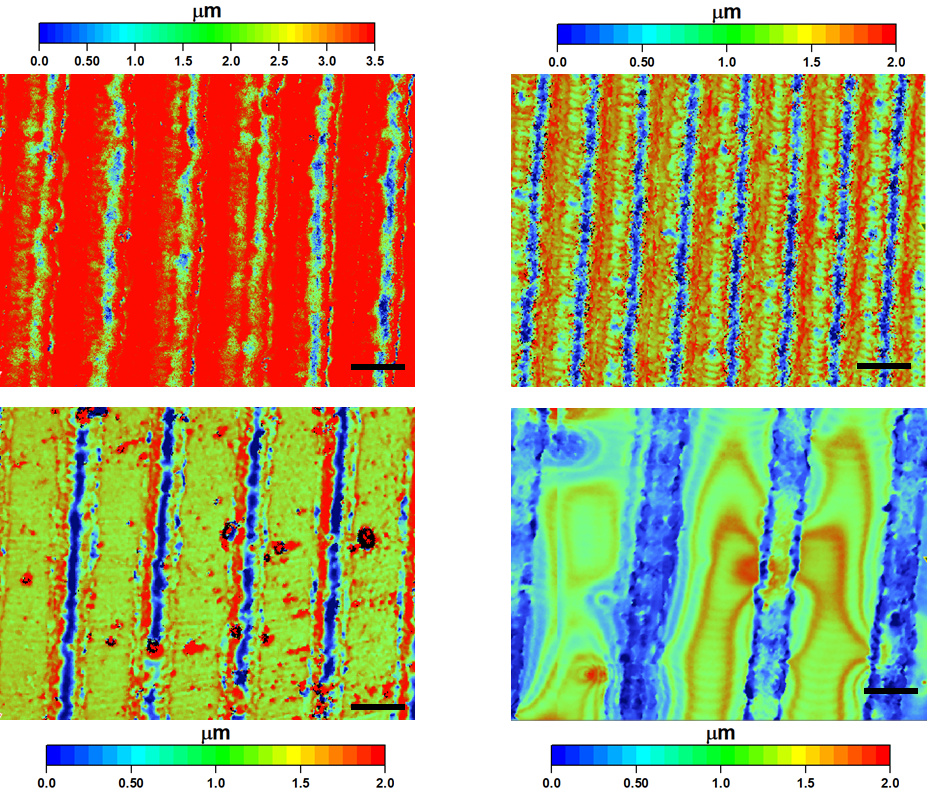
A Bruker Nano Contour GT-K Optical Profilometer was used to quantify the surface terrains. The surface profiles of surfaces 2-5 are presented in **Fig. S4**. The surface profile of sample surface 1 is provided in **Fig. 3f** of the main manuscript.

**(a)**

**(b)**

**(d)**

**(c)**

**Figure S4:** (a)-(d): Surface texture elevation profiles of surfaces 2-5 (**Table S1**), respectively, as obtained via optical profilometry. The color scale of (a) is different than that of (b)-(d).

*Glass Hydrophobization*

The glass samples were hydroxylated via oxygen plasma exposure (PPE-75, Plasma Etch, United States) for 30 minutes with a power of 40 W, and then exposed to (heptadecafluoro-1,1,2,2-tetrahydrodecyl)dimethylchlorosilane (Gelest, United States) vapor for 3 days at 75 °C or exposed to 1,3-dichlorotetramethyldisiloxane (Gelest, United States) vapor overnight.

*Copper Hydrophobization*

The copper samples were immersed in liquid soldering flux (Rubyfluid, American Beauty) to remove natural oxide, and then hydroxylated via oxygen plasma exposure (PPE-75, Plasma Etch, United States) for 30 min with a power of 40 W. Ultimately, the hydroxylated copper samples were soaked in 0.5 vol% 1H,1H,2H,2H-perfluorodecanethiol (Sigma Aldrich, United States) ethanol solution overnight in a sealed container with an argon-rich atmosphere.

*Derivation of Equation 4*

The increase of interfacial toughness mainly stems from the increase of contact area, giving

$\frac{Г_{⫽}}{Г_{0}}=r.$

For a sinusoidal interface, morphology can be described as

$f(x)=A\sin\left( \frac{2\pi}{\lambda}x \right).$ (S1)

Therefore, the Wenzel roughness *r* can be expressed as the arc length of the sine wave over the spacing λ, giving

$r=\frac{\int_{0}^{\lambda} \sqrt{1+{f^{'}(x)}^{2}}dx}{\lambda}=\frac{\int_{0}^{\lambda} \sqrt{1+\left( \frac{2\pi A}{\lambda} \right)^{2}\cos^{2} \left( \frac{2\pi}{\lambda}x \right)}dx}{\lambda}.$ (S2)

Let $u=\frac{2\pi}{\lambda}x$, then

$r=\frac{\frac{\lambda}{2\pi}\int_{0}^{2\pi} \sqrt{1+\left( \frac{2\pi A}{\lambda} \right)^{2}\cos^{2} \left( u \right)}du}{\lambda}=\frac{1}{2\pi}\int_{0}^{2\pi} \sqrt{1+\left( \frac{2\pi A}{\lambda} \right)^{2}\cos^{2} \left( u \right)}du.$ (S3)

Since $\cos^{2} \left( u \right)=1-\sin^{2} \left( u \right)$, we have

$r=\frac{1}{2\pi}\int_{0}^{2\pi} \sqrt{1+\left( \frac{2\pi A}{\lambda} \right)^{2}-\left( \frac{2\pi A}{\lambda} \right)^{2}\sin^{2} \left( u \right)}du=\frac{\sqrt{1+\left( \frac{2\pi A}{\lambda} \right)^{2}}}{2\pi}\int_{0}^{2\pi} \sqrt{1-\frac{\left( \frac{2\pi A}{\lambda} \right)^{2}}{1+\left( \frac{2\pi A}{\lambda} \right)^{2}}\sin^{2} \left( u \right)}du.$ (S4)

Using the definition of the complete elliptic integral of the second kind
$E\left( m \right)=\int_{0}^{\frac{\pi}{2}} \sqrt{1-m\sin^{2} x}dx$, we obtain **Equation 4** of the main paper
 $\frac{Г_{⫽}}{Г_{0}}=r=\frac{2}{\pi}\sqrt{1+4\pi^{2}\left( \frac{A}{\lambda} \right)^{2}}E\left( \frac{4\pi^{2}\left( \frac{A}{\lambda} \right)^{2}}{1+4\pi^{2}\left( \frac{A}{\lambda} \right)^{2}} \right)$. (4)

*Simulation*

The simulation of the fracture process across the trenches was conducted in COMSOL Multiphysics^®^. The Structural Mechanics Module was utilized to simulate fracture behavior in a study of “stationary” and 2D geometry. Ice of 1 mm height and 1 cm long (Density: 917 kg/m^3^; elastic modulus: 8.5 GPa; Poisson ratio: 0.3) was placed on top of a glass of the same dimensions (built-in properties). The pre-crack was 2 mm long, and “contact nodes” were applied to the rest of the ice-glass interface of 8 mm. The tensile and shear adhesion strengths were set as 228 kPa, and the tensile and shear adhesion strengths were set as 0.3 J/m^2^, similar to the experimental values. The boundary condition at the bottom of the glass is a “fixed constraint,” while other boundaries are “free” except for the one where the force is applied. Sinusoidal interfaces with varying amplitudes (A: 0, 0.25, 1, and 5 μm) and wavelengths (λ: 50, 100, and 200 μm) were applied. The longer side of the ice crossed through multiple trenches. By varying the displacement at one side of the ice force, we recorded the responding force and obtained the maximal force $\tilde{F}$ at the onset of detachment. The interfacial toughness can then be obtained by $Г_{\perp}=\frac{{\tilde{F}_{c}}^{2}}{2E_{ice}h_{ice}}$. $Г_{0}$, the interfacial toughness with A = 0 μm, was used to normalize both $Г_{\perp}$ and $Г_{⫽}$.

*Ice Adhesion Testing*

The measurements of $\hat{\tau}$ were conducted by the push-off test of ice [1]. The setup is shown in **Figure S5**. The size and shape of adhered ice were controlled using rectangular molds of dimensions 1-14 cm × 1 cm × 0.6 cm. In total, a minimum of three measurements per surface (*N* = 3) were taken at each ice length. The Peltier plate surface temperature was held at -10°C during testing. The shear force required to dislodge the ice was recorded using a force gauge (Nextech DFS500) moving at a controlled speed of 73.5 μm/s. For the icing cycle durability test, we fixed the ice length at 8 cm and conducted icing-deicing at three positions for 20 cycles. In the force-time curves, we fixed the ice length at 4 cm and tracked the force-time curve.


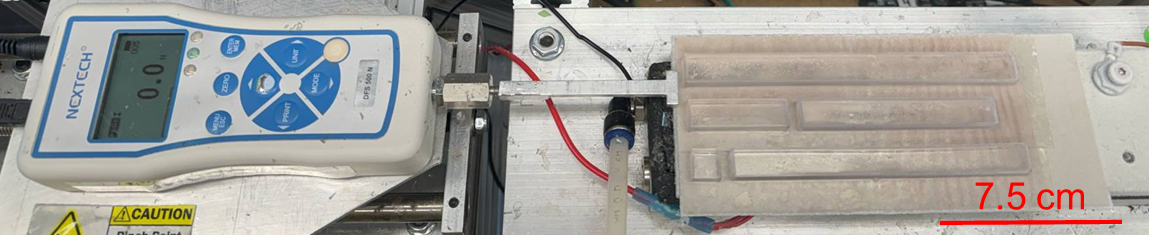


**Figure S5:** An image of the setup of the ice push-off tests. Ice slabs of different lengths (1 cm, 4 cm, 8 cm, 12 cm, and 14 cm, as shown at right) were placed on the test sample. A force gauge positioned on the left was used to dislodge the ice and record the peak force.

*Contact Angle Measurement*

Contact angle measurements were conducted using a Ramé-Hart 200-F1 contact angle goniometer via the sessile drop method. Advancing (ACA) and receding (RCA) contact angles were determined by gradually increasing or decreasing the volume of the test liquid drop and recording the corresponding angles at steady contact-line speed.

**
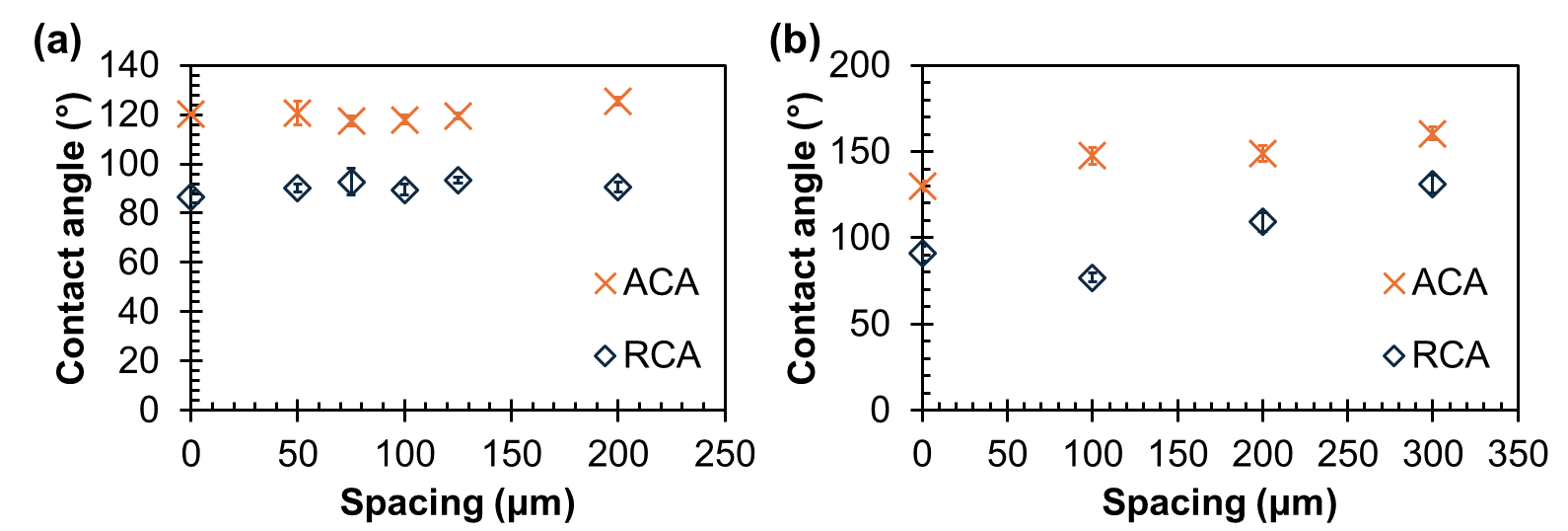
**

**Figure S6:** The advancing contact angles (ACA) and receding contact angles (RCA) of water on laser-etched, fluorinated (a) glass, and (b) copper with different roughness trench spacings. Substrates indicated with 0 μm spacing represent smooth (unprocessed) fluorinated substrates.

**
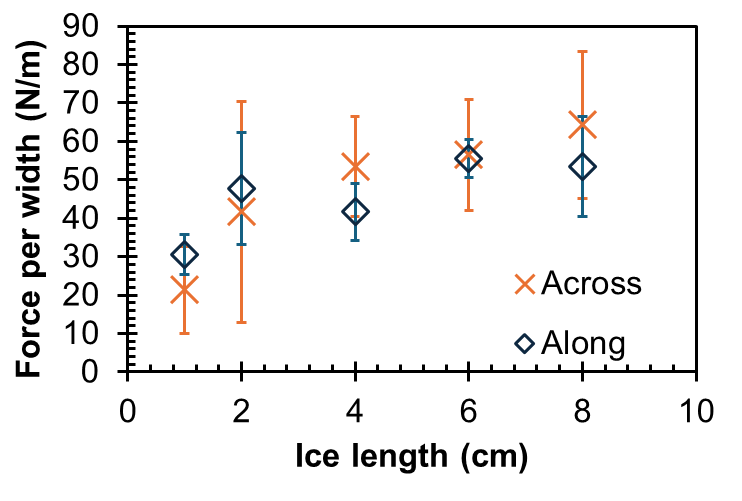
**

**Figure S7:** Measured force per unit width vs. ice length values for 1,3-dichlorotetramethyldisiloxane grafted, etched glass with trench spacing of 50 μm with different ice/roughness relative orientations. “Across” denotes that cracks propagate across multiple trenches. “Along” denotes that cracks propagate along the trenches.


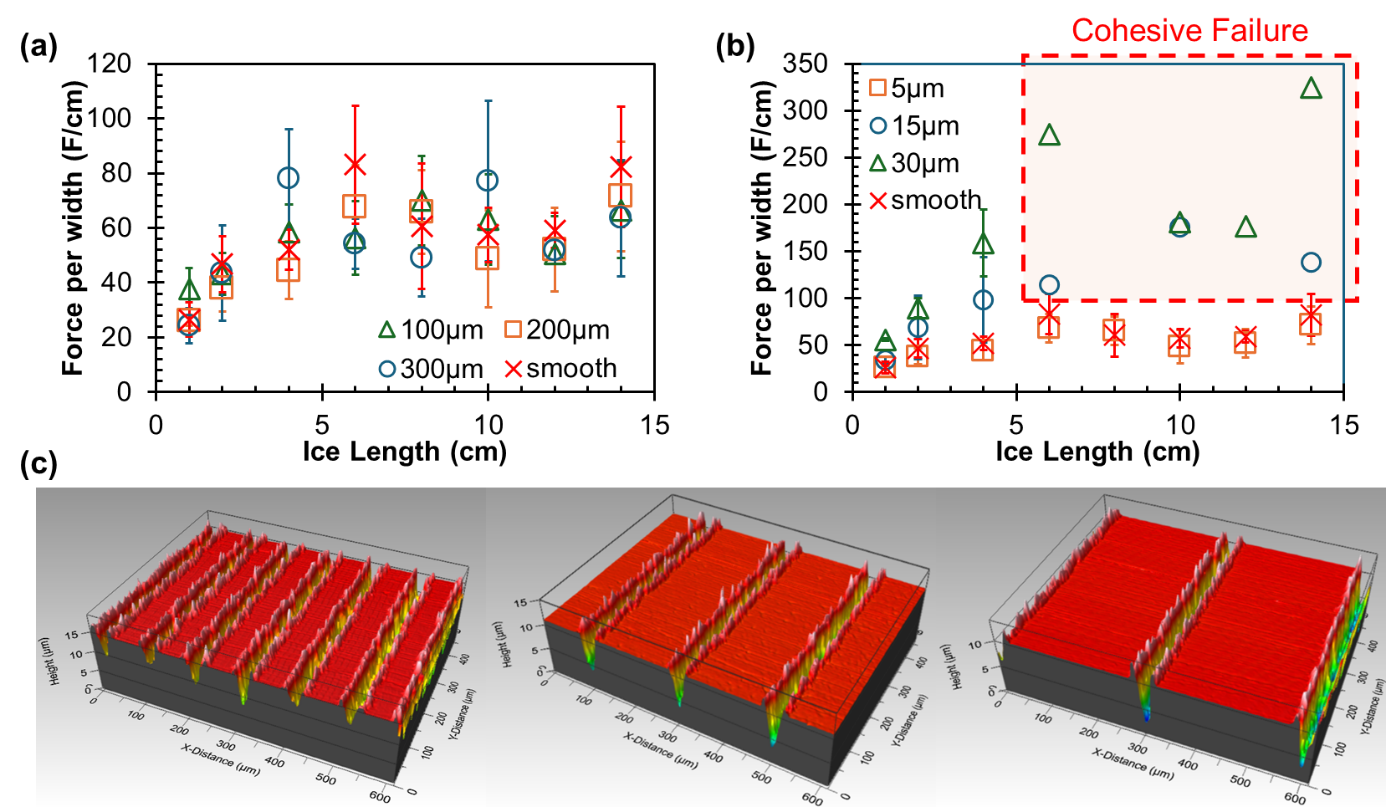


**Figure S8:** Ice shedding performance and topography of etched copper. (a) Force per width vs. ice length data on etched copper with different trench spacings and a depth of 5 μm. (b) Force per width vs. ice length curves of etched copper with different depths and a trench spacing of 200 μm. The “15 μm” and “30 μm” cases encountered cohesive failure at ice lengths greater than 4 cm. The error bars in (a) and (b) denote the standard deviation among at least three measurements. (c) Topography of etched copper with different trench spacings (100 μm, 200 μm, and 300 μm from left to right).

**Reference**

*1.* Golovin, K., Dhyani, A., Thouless, M. D., Tuteja A., Low-interfacial toughness materials for effective large-scale deicing*. Science* **364**: pp. 371-375 (2019).
